# Patient-centered benefit-risk analysis of transcatheter aortic valve replacement

**DOI:** 10.12688/f1000research.18796.5

**Published:** 2021-02-08

**Authors:** Kevin Marsh, Natalia Hawken, Ella Brookes, Carrie Kuehn, Barry Liden

**Affiliations:** 1 Patient-Centered Research, Evidera Inc, London, UK; 2 Modus Outcomes, London, UK; 3 Patient Engagement, Edwards Lifesciences, Washington D.C., USA

**Keywords:** TAVR, aortic valve, transcatheter, patient preference, benefit-risk analysis

## Abstract

**Background**: Aortic stenosis (AS) treatments include surgical aortic valve replacement (SAVR) and transcatheter aortic valve replacement (TAVR). Choosing between SAVR and TAVR requires patients to trade-off  benefits and risks. The objective of this research was to determine which  TAVR and SAVR outcomes patients consider important, collect quantitative data about how patients weigh benefits and risks, and evaluate patients’ preferences for SAVR or TAVR.

**Methods**: Patients  were recruited from advocacy organization databases. Patients self-reported as being diagnosed with AS, and as either having received AS treatment or as experiencing AS-related physical activity limitations. An online adapted swing weighting (ASW) method – a pairwise comparison of attributes – was used to elicit attribute trade-offs from 219 patients. Survey data were used to estimate patients’ weights for AS treatment attributes, which were incorporated into a quantitative benefit-risk analysis (BRA) to evaluate patients’ preferences for TAVR and SAVR.

**Results**: On average, patients put greater value on attributes that favored TAVR than SAVR. Patients’ valuation of the lower mortality rate, reduced procedural invasiveness, and quicker time to return to normal quality of life associated with TAVR, offset their valuation of the time over which SAVR has been proven to work. There was substantial heterogeneity in patients’ preferences. This was partly explained by age, with differences in preference observed between patients <60 years to those ≥60 years. A Monte Carlo Simulation found that 79.5% of patients prefer TAVR.

**Conclusions**: Most AS patients are willing to tolerate sizable increases in clinical risk in exchange for the benefits of TAVR, resulting in a large proportion of patients preferring TAVR to SAVR. Further work should be undertaken to characterize the heterogeneity in preferences for AS treatment attributes. Shared decision-making tools based on attributes important to patients can support patients’ selection of the procedure that best meets their needs.

## Introduction

Aortic stenosis (AS) is a progressive cardiovascular condition resulting from narrowing (or stenosis) of the aortic valve. This narrowing prevents the valve from fully opening, decreasing blood flow out of the heart and forcing it to work harder to maintain sufficient blood flow. If left untreated, AS can lead to severe cardiovascular complications and death ^1^. As of 2015, 12.4% of the United States population over age 75 (nearly 2.5 million people) were reportedly diagnosed with AS ^2, 3^. More than one in eight people (13.3%) over the age of 75 in the US have moderate-to-severe AS ^4^. Patients with AS may be asymptomatic for many years until the valve is narrowed severely enough to cause symptoms. Symptoms of AS include chest pain and angina, syncope, dyspnea, fatigue, and palpitations, all of which are exacerbated by physical activity ^5^. Undertreatment of AS patients is common, with more than half of patients referred to cardiologists failing to receive surgical treatment ^6^. Once symptoms appear, often between the ages of 70 and 80 years old, the prognosis of untreated patients is poor ^7^. Among untreated patients, average survival is 50% at two years and 20% at five years after the onset of AS symptoms.

The traditionally recommended treatment for AS is surgical aortic valve replacement (SAVR). Such invasive surgery, involving a large incision in the chest, may not be suitable for all patients, especially those with comorbidities. The alternative, transcatheter aortic valve replacement (TAVR), is a minimally invasive, catheter-based procedure to replace the aortic valve in patients with AS. TAVR is the first-line therapy for inoperable patients with severe AS and an alternative to SAVR in operable high-risk patients. Among patients who are at intermediate surgical risk, both TAVR and SAVR are associated with improvements in disease-specific and generic health status ^8^. However, TAVR is associated with a reduced rate of complications and a quicker recovery time, with patients returning to a normal quality of life more quickly ^9^. When first available, the benefits of TAVR were offset by reportedly increased risks of stroke and the need for a pacemaker ^10– 12^. Recent clinical data reveal similar, if not improved rates of stroke and need for pacemaker among TAVR patients ^13^. Furthermore, at a median two-year follow-up, all-cause mortality for patients undergoing TAVR was 20.2% compared with 21.9% for patients undergoing SAVR ^14^.

The choice of TAVR or SAVR involves patients making trade-offs between multiple treatment attributes, including invasiveness, speed of recovery, mortality rates, and risks of complications. Given the challenging nature of this decision, tools have been developed to support patient decision-making ^15^. However, little is known about the weights that patients assign to each attributes, how they make trade-offs between these attributes, and whether and how these preferences vary between patients. The objective of this research was to determine which outcomes associated with TAVR and SAVR patients consider most important, collect quantitative data about how patients weigh the benefits and risks associated with TAVR and SAVR, and to use this data to evaluate patients’ preferences for SAVR or TAVR.

Interim results from the first 93 participants enrolled into this study were published on 21 June 2019 as Version 3. This manuscript reports the final results from this study, based on a sample of 219 participants.

## Methods

### Overview

Patients’ preferences for TAVR or SAVR were assessed using a quantitative benefit-risk assessment (BRA). This involved identifying attributes that distinguish TAVR and SAVR, measuring TAVR and SAVR performance on these attributes, eliciting patients’ preferences for these attributes, and combining performance and preference in a BRA to determine what proportion of patients prefer TAVR or SAVR. The patient-preference survey upon which the BRA is based was fielded from July 2018 – January 2019.

### Attribute selection

A long list of potential attributes was identified by reviewing the attributes highlighted in previous patient preference studies for heart valve surgical interventions, published meta-analyses and clinical studies, and regulators’ assessments of related products. Additional attributes were identified based on consultation with TAVR and SAVR clinical experts and from patient input. The final attributes used in the study were selected based on clinical and regulatory relevance, whether or not the attribute distinguishes between TAVR and SAVR, and to comply with the attribute set properties required of an additive BRA ^16^. For example, to avoid overlap with ‘mortality’, the ‘stroke’ attribute was defined as ‘disabling, non-fatal strokes.’

Descriptions of the final attributes included in the BRA are summarized in Table 1.

**Table 1.** Selected attributes for the patient-centered benefit-risk analysis of transcatheter aortic valve replacement vs surgical aortic valve replacement.

Attribute	Definition	Description provided to participants
Type of procedure	Type of procedure	The invasiveness of the procedure is described by three characteristics: The length and depth of the incision Whether you heart is stopped The number of days that you will need to be in the hospital following a procedure. There are two types of procedure: A minimally invasive procedure requiring, on average, 8 days in hospital. A small incision is made near your groin, and a valve is inserted and guided to your heart using a long tube through an artery. The tube is used to implant a new valve in the heart to replace the diseased aortic valve. An invasive procedure, requiring, on average, 12 days in hospital. A large cut about 25 cm long is made in your chest to access your heart. Then, your heart is stopped while a machine takes over your heart and lung function. A new valve is implanted to replace the damaged valve. Your heart is started again, and your chest is stitched closed.
Mortality	Number of patients out of 100 who will die within 1 month	The numbers of patients who will die from any cause within 1 month of having the procedure. Death could be due to complications from the procedure, from complications of aortic stenosis, or as a result of disabling stroke.
Disabling non- fatal stroke	The number of patients out of 100 who will experience a non-fatal disabling stroke within 1 month	The number of patients who will experience a non-fatal but disabling stroke within 1 month of having the procedure. If you experience a stroke, you will be hospitalized. If the stroke is severe, it may lead to temporary or permanent disability, such as paralysis, reduced mobility, and problems with thinking, memory and speech.
Independence	Number of patients out of 100 who experience greater independence within 1 month	The number of patients who experience improvement in daily activities (greater independence) following relief from aortic stenosis symptoms within 1 month of the procedure. The symptoms of aortic stenosis (shortness of breath, fatigue, chest pain, and dizziness), physical function, and quality of life are improved to an extent that you experience improvements in your degree of independence and ability to engage in activities of daily living.
New permanent pacemaker	The number of patients out of 100 who will require a pacemaker within 1 year	The number of patients that will need to have a pacemaker permanently implanted as a result of the procedure. Typically, a pacemaker is implanted under local anesthetics and you may be discharged the same day if you get your pacemaker in the morning.
Requirement for dialysis	The number of patients out of 100 who will require dialysis within 1 year	The number of patients that will experience kidney function damage that will need dialysis as a result of the procedure. A machine is used to do the kidney’s job of cleaning the blood. If you need dialysis, you will need to go to the hospital three times a week, with each visit lasting 4 hours.
Time over which the procedure has been proven to work	The number of years for which your procedure has been available and proven to work	The number of years the procedure has been available and is proven to keep symptoms of aortic stenosis from coming back. Following this period, it is currently not known whether you will experience aortic stenosis symptoms again.

### Performance measurement

TAVR and SAVR performance against the final attributes were identified from the published literature and from clinical data ( Table 2), focusing on data that had a high degree of reference and use within the clinical community. Available data for stroke risk, defined as “all stroke” (both fatal and non-fatal) in the literature, and independence, defined by Kansas City Cardiomyopathy Questionnaire (KCCQ) ^17^ score, required transformation to estimate performance as defined by the final study attributes. To estimate the risk of non-fatal stroke only, available stroke risk data was adjusted using the mortality rate among patients with severe aortic stenosis enrolled in the PARTNER trial who suffered a stroke compared to the mortality rate among those in the trial who did not suffer a stroke ^18^. ‘Independence’ was defined as the probability of achieving relief from AS symptoms within a month of a procedure. Given available data, this was estimated as the probability of achieving a total score of 75 on KCCQ ^17^. The KCCQ is a standard patient reported outcome measure used in clinical trials of surgical and transcatheter heart valves ^21^. Estimated mean KCCQ score and variation in this measure were transformed into the proportion of patients achieving a KCCQ total score of 75 at 1 month using procedures previously described in Marchini ^22^.

**Table 2.** Transcatheter aortic valve replacement (TAVR) and surgical aortic valve replacement (SAVR) performance against benefit-risk analysis attributes.

Attribute	Measure	TAVR		SAVR		Performance Range
		Mean	95% CI	Mean	95% CI	
Mortality (all cause) ^19^	One-month risk	0.011	0.005-0.017	0.040	0.028-0.053	0.005-0.053
Disabling non-fatal stroke ^19^	One-month risk	0.008	0.002-0.013	0.033	0.021-0.044	0.002-0.044
Independence ^8^	Probability of having relief from AS symptoms that have an effect on daily life	0.479	0.454-0.500	0.249	0.227-0.276	0.227-0.500
New permanent pacemaker ^19^	One-year risk	0.123	0.103-0.142	0.090	0.072-0.108	0.072-0.142
Requirement for dialysis ^20^	One-year risk	0.032	0.021-0.042	0.047	0.034-0.060	0.021-0.060
Time over which the Procedure has been Proven to Work	Years	10		20		5 - 30

CI, confidence interval; AS, aortic stenosis.

### Survey methodology

**Figure 1.**
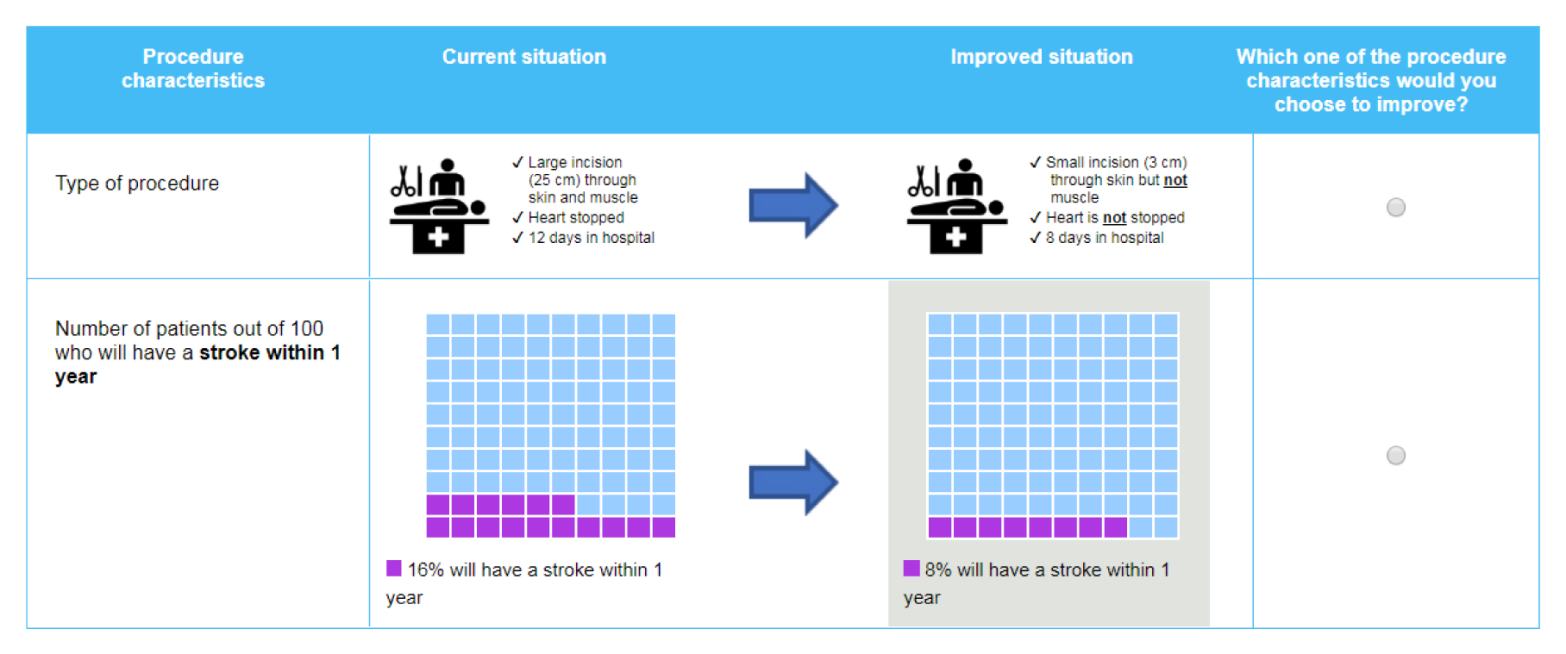
Example adapted swing weighting question presented to study participants. **‘**Type of Procedure’ was used in every pairwise comparison. Only the comparison attribute was varied across different attributes and performance levels.

An adapted swing weighting (ASW) exercise was administered online to elicit patients’ preferences for treatment attributes ^23^. The objective of the ASW exercise was to identify the level of change in an attribute that patients would be willing to accept in exchange for improving their procedure from ‘invasive’ to ‘minimally invasive’ (see Table 1 for definitions). The ASW exercise consisted of a series of pairwise comparisons of attributes—the ‘invasiveness’ attribute and one other attribute. Participants were shown ‘current’ and ‘improved’ levels on each attribute and asked which improvement they would prefer to make (an example choice question is shown in Figure 1). The ‘current’ levels were chosen to reflect the attribute performance levels of TAVR and SAVR ( Table 2), to ensure they had credibility with patients, adjusted to ensure that patients had sufficient range to indicate the change in the attribute that would have the equivalent value as reducing invasiveness. Therefore, the exercises were not designed to directly elicit patients’ willingness to tolerate the actual change observed with TAVR.

Each set of pairwise comparisons included three iterations of the choice question. Depending on the answer to the choice question, the level of improvement offered on the non-procedure attribute was updated in the direction that made the value of improvements on the two attributes more similar than in the previous question. The algorithm used to identify participants’ indifference point included the levels used in each of the three iterations and how these changed dependent on the participants’ previous responses is shown in extended data, Appendix 1 ^24^.

A pilot of the survey among five AS patients, carried out over a 4-week period in June 2018, ensured acceptable cognitive burden, clarity of the instructions, and ease of use of the elicitation software. Before completing the ASW exercises, participants were introduced to the attributes and their definitions. Participants also completed two ASW practice questions; interpretation of their response to these practice questions was tested to ensure participants understood how to complete each of the pairwise comparisons. Only eighteen participants (8.2%) incorrectly interpreted the meaning of their response to the first practice question, and no participants incorrectly interpreted their response to the second practice question. Each participant completed a proportion of the possible pairwise question—either 3 or 4 sets of pairwise comparison questions. Participants also completed clinical and demographic questions, and health literacy and numeracy questionnaires, available as extended data ^24^.

### Participants

Potentially eligible participants were recruited by M3 Global Research via email and from the membership of Heart Valve Voice and Mended Hearts patient organizations through e-mails and advertisements on social media platforms. Patients from the American Heart Association membership who had given prior permission to receive mailings were also invited to participate. Potential participants were directed to an online screening tool, where their eligibility for the survey was determined. Participants had to meet specific inclusion/exclusion criteria to participate ( Box 1). There were no predefined enrollment targets or stratification by other demographic characteristics. Following completion of the online screening tool, eligible participants were directed to the web survey. They were also sent a link to the web survey, available as extended data ^24^, via e-mail so that they were able to complete the study at a time that was convenient for them.

Box 1. **Eligibility criteria**

**Inclusion criteria**

- Be at least 19 years of age
- Have a self-reported diagnosis of aortic valve disease
- Able to read and understand English
- Willing and able to complete an online survey
- Willing and able to provide (electronic) consent to participate in study
- Be a resident of the United States
- Have been treated procedurally (TAVR or SAVR) for AS in the past 10 years

or

- If untreated, must have experienced at least one of the following symptoms of AS:
  ■ Breathlessness/Shortness of breath
  ■ Tiredness/Lack of energy/Fatigue
  ■ Chest pain
  ■ Dizziness or blackouts/Syncope

- Be willing and able to participate in a telephone interview, and to be audio-recorded (qualitative pilot only)

**Exclusion criteria**

- Have a cognitive impairment, hearing difficulty, visual impairment, acute psychopathology
- Have insufficient knowledge of English, which could interfere with the patient’s ability to provide written consent and complete the web survey
- Are not experiencing at least one of the symptoms of AS, as described in the inclusion criteria

### Analysis

Participant responses to the ASW questions identified the level of change in an attribute that patients would be willing to accept in exchange for improving their procedure from ‘invasive’ to ‘minimally invasive.’ For instance, a participant might assign the same value to a 2% reduction in mortality risk as reducing the invasiveness of the procedure. Inverting this relationship, responses were used to estimate the maximum acceptable increase in risks (MIR) or the maximum acceptable reduction in benefits (MRB) that participants would be willing to tolerate in exchange for moving from an invasive procedure to a minimally invasive procedure. In the above example, the participant would be willing to tolerate a MIR of 2% in mortality risk to reduce the invasiveness of the procedure.

In cases of incomplete or missing data, no data imputation was performed, and unanswered questions were coded as missing. Data from participants who completed only a portion of the survey were included in the analyses if they had completed all the questions in at least one ASW exercise.

Participant preferences were incorporated into the following benefit-risk model constructed in Microsoft Excel.

$$U\text{(x)}=\sum_{i=\text{1}}^{n}w_{i}v_{i}(x_{i})\;(Equation 1)$$

Where

*U*( *x*) is the overall value generated by procedure *x*.
*w _i_* is the weight associated with attribute *i*,
*v _i_* is the partial value function for attribute *i*, which was assumed to be linear
*x _i_* is the performance of performance *x* on attribute *i*

Participants’ MIR/MRB was converted into an attribute weight ( *w _i_*) by setting the weight for ‘type of procedure’ to 1, and then dividing the range in performance between TAVR and SAVR on that attribute ( Table 2) by the MIR/MRB. For instance, if patients were willing to tolerate a 2% increase in mortality risk in exchange for a reduction in invasiveness, and reducing invasiveness is given a weight of 1, then the 4.8% change in mortality risk covered by the benefit-risk model (0.5%–5.3% range identified in Table 2) would be given a weight of 2.4 (4.8%/2%). Weights across all attributes in the model were then normalized to sum to 100.

Four outputs were generated from the benefit-risk model to evaluate patients’ preferences for TAVR and SAVR. First, the incremental overall value generated by TAVR:

U(increment)=U(TAVR)−U(SAVR)(Equation 2)

Second, the incremental partial value on each attribute generated by TAVR:

$$PV_{i}(increment)=w_{i}v_{i}(x_{TAVR})-w_{i}v_{i}(x_{SAVR})\;(Equation 3)$$

Where

*x _TAVR_* is the performance of TAVR on attribute *i*
*x _SAVR_* is the performance of SAVR on attribute *i*

Third, a threshold analysis was undertaken, estimating the level of performance (minimum acceptable benefit or maximum acceptable risk) on each attribute that would leave patients indifferent between TAVR and SAVR. More specifically:

$$MAR_{a}\;or\;MAB_{a}=TAVR_{a}+((U(TAVR)-U(SAVR))/w_{i}v_{i}(x_{1\;unit\;a})\;(Equation 4)$$

Where

*MAR _a_* or *MAB _a_* is the maximum acceptable risk or minimum acceptable benefit of attribute *a*
*TAVR _a_* is the performance of TAVR on attribute *a* (see Table 2).
*x*_1_ *_unit a_* is a single unit of performance on attribute *a*

Table 3 outlines the definitions of MIR, MRB, MAR, and MAB.

**Table 3.** Benefit-risk analysis definitions.

Outcome	Definition
**MIR**	**Maximum Acceptable Increase in Risk:** The maximum acceptable increase in risk for a single attribute that patients would tolerate in exchange for reducing procedure invasiveness.
**MRB**	**Maximum Acceptable Reduction in Benefit:** The maximum acceptable reduction in benefit for a single attribute that patients would tolerate in exchange for reducing procedure invasiveness.
**MAR**	**Maximum Acceptable Risk:** The maximum acceptable risk that would make patients indifferent between TAVR and SAVR.
**MAB**	**Minimum Acceptable Benefit:** The minimum acceptable benefit that would make patients indifferent between TAVR and SAVR.

TAVR, transcatheter aortic valve replacement; SAVR, surgical aortic valve replacement.

Fourth, a Monte Carlo Simulation (MCS) was run to explore uncertainty in model inputs. That is, the benefit-risk model was run 10,000 times for both TAVR and SAVR. In each instance, the model drew from the distribution around both performance and weight inputs. Specifically, performance inputs were drawn from the distribution around TAVR and SAVR performance data ( Table 2). Weight inputs were drawn in a manner that reflected the probability that participants identified different MIR/MRBs in their responses to the survey. For each iteration of the MCS, TAVR and SAVR, we’ve ranked based on *U* ( Equation 2) and the proportion of instances that TAVR ranked first was estimated.

### Ethics

In accordance with ethical practice, Institutional Review Board (IRB) approval was obtained through Advarra (approval MOD00300398 and MOD00354863) to comply with human participants research requirements prior to initiation of participant recruitment or administration of measures in the pilot or main studies. Informed consent was recorded electronically via the online survey platform. Any change to the protocol and/or informed consent form was resubmitted to the IRB for review and approval prior to implementation. The study was available for monitoring, auditing, IRB review, and regulatory inspection as applicable.

## Results

### Demographic characteristics of participants

A total of 219 patients completed the survey over two rounds ( Table 4). Raw data are available from Open Science Framework ^24^. The majority of patients were less than 60 years old (n=132, 60.3%). More than half of the respondents were female (n=128, 58.4%) and a majority were white (n=173, 79.0%). Over half of the sample had completed a college degree or higher (n=157, 71.7%). Most of the participants lived with a partner/spouse, family, or friends (n=164, 74.9%). No participants reported experiencing severe limitations to their physical activity. Few patients demonstrated low health literacy (n=23, 10.5%) or numeracy (n=22, 10.0%), and only 7 patients (3.2%) were low on both scales.

**Table 4.** Demographic and clinical characteristics of aortic stenosis patients who completed the preference elicitation survey (N=219).

Characteristics	Patients, n (%)
Gender	
Male	91 (41.6%)
Female	128 (58.4%)
Ethnic background	
Not Hispanic or Latino	209 (95.4%)
Age group, years	
19–39	58 (26.5%)
40–59	74 (33.8%)
60–74	55 (25.1%)
75–89	29 (13.2%)
90+	3 (1.4%)
Racial background	
White	173 (79.0%)
Black or African American	27 (12.3%)
Asian	13 (5.9%)
Native Hawaiian or other Pacific Islander	3 (1.4%)
American Indian or Alaskan Native	4 (1.8%)
Other	7 (3.2%)
Living Situation	
Living alone	54 (24.7%)
Living with a partner or spouse, family, or friends	164 (74.9%)
Employment Status	
Employed, full-time	93 (42.5%)
Employed, part-time	28 (12.8%)
Homemaker	8 (3.7%)
Student	6 (2.7%)
Unemployed	8 (3.7%)
Retired	71 (32.4%)
Disabled	19 (8.7%)
Highest level of education completed	
Secondary/high school	16 (7.3%)
Some college	46 (21.0%)
College degree	93 (42.5%)
Postgraduate degree	63 (28.8%)
Other:	1 (0.5%)
General health within past week	
Very good	55 (25.1%)
Good	85 (38.8%)
Fair	65 (29.7%)
Poor	13 (5.9%)
Very poor	1 (0.5%)
Previously treated for AS	
Yes	176 (80.4%)
No	43 (19.6%)
NYHA Classification	
No limitation of physical activity	78 (35.6%)
Slight limitation of physical activity	101 (46.1%)
Marked limitation in physical activity	40 (18.3%)
Severe limitation in physical activity	0 (0.0%)
Health literacy and numeracy	
Low literacy ( 1)	23 (10.5%)
Low numeracy ( 2)	22 (10.0%)
Overall low literacy / numeracy ( 3)	7 (3.2%)

^1^Responses were scored between 0 (always) and 4 (never). Each participants’ scored responses were averaged for a composite score ranging from 0–4. A low score if ≤2. ^2^Participants were given one point for each correctly answered question (maximum numeracy score = 5). A low score if given is ≤2 answered incorrectly. ^3^Overall low: individuals who scored low on both educational level and objective health literacy. NYHA, New York Heart Association.

### Responses to ASW questions

When responding to the ASW questions, only a small proportion (8.68%) of participants ‘straight-lined’ on all questions—consistently choosing to improve either ‘procedure’ or the comparison attribute across all three iterations of the choice question. These responses may be a valid reflection of participants’ preference—suggesting a strong preference either for avoiding an invasive procedure, or a strong preference to prioritize improving other procedure attributes. Thus, these responses were included in the analysis. The impact of excluding these data were tested, and it was determined that results of the BRA were not sensitive to whether these data were included or excluded.

### Comparisons of TAVR and SAVR

Table 5 shows the difference in performance of TAVR compared with SAVR on each attribute, and patients’ willingness to accept this difference in exchange for the lesser invasiveness of TAVR. The increase in risks or the reduction in benefits that patients are, on average, willing to accept in exchange for reducing procedure invasiveness is reported in the middle three columns. For instance, patients would be willing to tolerate a 6.69% increase in the probability of experiencing disabling, non-fatal stroke in exchange for the reduction in invasiveness associated with receiving TAVR instead of SAVR. In each case, patients were on average willing to accept TAVR’s performance on any attribute in exchange for its lower invasiveness. In the case of four attributes (mortality, disabling non-fatal stroke, independence, and dialysis), TAVR performs better than SAVR. Where SAVR performs better than TAVR (the need for new permanent pacemaker and time over which the procedure has been proven to work), patients would, on average, be willing to accept TAVR’s performance given its lower invasiveness. For example, participants are willing to tolerate a 6.98% increase in the risk of a new permanent pacemaker, while the probability of needing a new permanent pacemaker only increases by 3.3% with TAVR.

**Table 5.** Patients Maximum Acceptable Increase in Risk (MIR)/Minimum Acceptable Reduction in Benefit (MRB) in Exchange for Reducing Procedure Invasiveness from ‘Invasive’ to ‘Minimally Invasive’.

Attribute	Impact of TAVR compared with SAVR †	Maximum Acceptable Increase in Risk/Maximum Acceptable Reduction in Benefit [Mean, (SD, n)]				Proportion of participants MIR/MRB > TAVR impact
		Whole sample	<60yrs old	≥60yrs old	p-value	
Mortality (1 Month)	-2.9%	3.86% (2.95, 109)	3.4 (2.7, 65)	4.5 (3.2, 44)	0.0521 *	100%
Disabling Non-Fatal Stroke	-2.5%	6.69% (5.73, 110)	6.5 (5.6, 67)	7.0 (6.0, 43)	0.6524	100%
Independence	+23.0%	13.94% (11.77, 131)	13.5 (11.5, 80)	14.6 (12.2, 51)	0.5949	100%
New Permanent Pacemaker	+3.3%	6.98% (5.71, 132)	5.9 (5.1, 76)	8.4 (6.2, 56)	0.0121 **	69.8%
Requirement for Dialysis	-1.5%	6.21% (5.62, 131)	5.4 (5.1, 81)	7.5 (6.2, 50)	0.0351 **	100%
Proven to Work	-10yrs	17.42 years (16.86, 131)	17.9 (16.7, 80)	16.6 (17.2, 51)	0.6668	47.3%

†See Table 2 for source of data * p<0.1, **p<0.05

The standard errors in patients’ MIR/MRB suggests a substantial heterogeneity in patients’ responses to the ASW exercise (see extended data, Appendix S2 ^24^ for more detail). Some of this heterogeneity was associated with participants’ age. MIR/MRB for three attributes—probability of having a new permanent pacemaker, probability of requiring dialysis, and one month mortality risk—were associated with whether patients are over or under 60 years old. Older patients were more willing to tolerate increases in risks/reductions in benefit to avoid having to undergo an invasive procedure.

No other correlation was found between participant characteristics and MIR/MRB. This includes whether a participant reported having previously undergone treatment for their AS. While this might be expected to influence preferences, the ability of the analysis to identify this influence is limited by the relatively small sample size and the small proportion of participants who reported not having previously received AS treatment (19.6%).

There were a large proportion of participants whose individual MIR/MRB was greater than the change in attribute performance achieved with TAVR ( Table 5). For attributes where performance is better with TAVR compared to SAVR (mortality, disabling non-fatal stroke, independence, and dialysis), 100% of patients would prefer the improved performance and reduced invasiveness of TAVR. For the two attributes on which attribute performance is better with SAVR compared to TAVR, 70% of patients would be willing to accept the increased risk of needing a new permanent pacemaker, and 47% of participants would be willing to accept the shorter period for which TAVR had been proven to work in order to experience TAVR’s reduced invasiveness.

**Figure 2.**
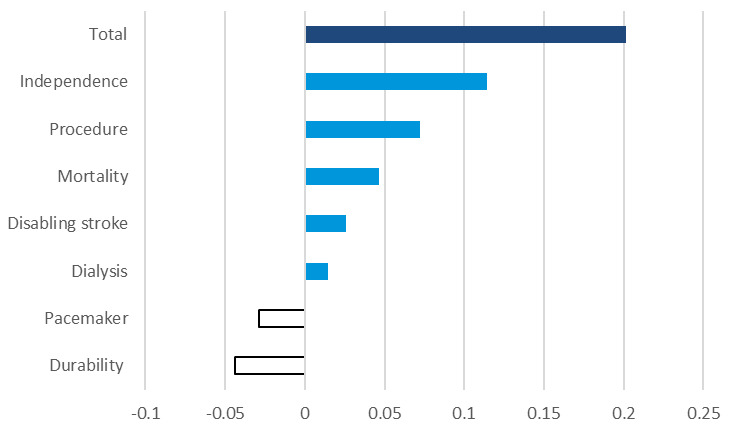
Incremental value of transcatheter aortic valve replacement vs. surgical aortic valve replacement in patients ≥60 years old.

**Figure 3.**
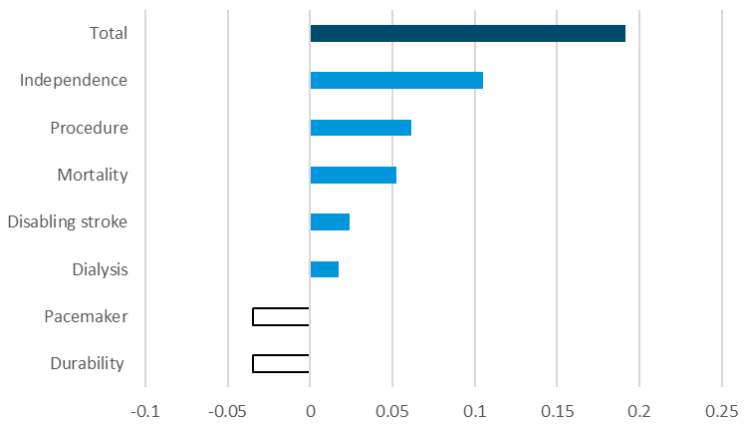
Incremental value transcatheter aortic valve replacement vs. surgical aortic valve replacement in patients <60 years old.

The above analysis compares the performance of TAVR on each attribute separately. A complete comparison of TAVR and SAVR should do so across all attributes simultaneously and take into account observed heterogeneity (in this case across age groups). This objective is accomplished by means of the benefit-risk model (see Equation 2 and Equation 3). Figure 2 and Figure 3 show the incremental value of TAVR (overall and by attribute) observed among patients 60 years old or older ( Figure 2) and among patients less than 60 years old ( Figure 3). These figures reveal that, overall, TAVR is of greater value to patients than SAVR. Specifically, patients placed greater value on TAVR based on a lower short-term mortality rate, reduced procedural invasiveness, and a quicker time to return to normal quality of life (independence) offsetting the value patients placed on longer period over which the procedure has been proven to work and reduced risk of needing a pacemaker generated by SAVR. Similar patterns were observed among younger and older patients.

### TAVR threshold analysis

Table 6 reports a threshold analysis, which shows the minimum amount of benefit that patients would accept before preferring TAVR, or the maximum amount of risk that patients would tolerate before preferring TAVR. For example, given the incremental value that patients attach to TAVR (as reflected in Figure 2 and Figure 3) they would be willing to tolerate a mortality risk of 12.6% following TAVR before they would be indifferent between TAVR and SAVR.

**Table 6.** Threshold Analysis.

	Minimum Acceptable Benefit/ Maximum Acceptable Risk			Outcome with TAVR
	Whole Population	<60 years old	≥60 years old	
Mortality (1 Month)	12.6%	11.6%	13.4%	1.1%
Disabling Non-Fatal Stroke	20.7%	21.0%	19.9%	0.8%
Independence	6.5%	6.0%	8%	47.9%
New Permanent Pacemaker	33.0%	30.6%	35.2%	12.3%
Requirement for Dialysis	21.6%	19.9%	23.7%	3.2%
Proven to Work	0yrs	0yrs	0yrs	10yrs

The MCS shows that 79.5% of patients would prefer TAVR over SAVR. When the analysis is run separately for patients less than and greater than 60 years old, the proportions of patients preferring TAVR are 80.8% and 78.2% respectively. Removing patients who ‘one-lined’ in response to ASW exercises does not impact the results of the MCS, with 80.7% of patients still preferring TAVR.

## Discussion

The choice between TAVR and SAVR for the treatment of AS involves making trade-offs between: procedure invasiveness; the period over which the procedure has been proven to be effective; mortality, stroke and independence outcomes; and risks such as the need for a new pacemaker or dialysis. This study elicited patients’ preferences for AS procedure attributes to determine how they make these trade-offs, and thus whether they prefer TAVR or SAVR. Results suggest that, given the potential benefits and risks of TAVR and SAVR, on average, patients attach more value to TAVR, and the majority of patients would prefer TAVR. Patients placed a greater value on the lower invasiveness, quicker speed of recovery, and reduced risk of mortality, stroke and need for dialysis associated with TAVR than they did on longer period over which the procedure has been proven to work and reduced risk of needing a pace maker associated with SAVR.

Current guidelines from the American Heart Association for the procedural treatment of AS do not take into account recent clinical data supporting the use of TAVR ^25^. Based on the recent clinical results of TAVR and the findings of this study, regulators may reach different conclusions about the need to protect patients from risks historically associated with TAVR. For instance, TAVR may not result in the increased risk of stroke that regulators might expect it to, and patients may be willing to tolerate the greater need for a permanent pacemaker in order to experience the benefits of TAVR.

The BRA revealed substantial heterogeneity in patient preferences for AS treatment. Some preference heterogeneity is explained by patient age, with older patients being less willing to tolerate the invasiveness of SAVR, instead preferring to accept greater potential risks associated with other procedure attributes in order to reduce the invasiveness. However, preference heterogeneity raises concerns about the attendance of participants to the preference elicitation tasks. A small proportion (8.68%) of participants straight-lined on all questions. While this might indicate a lack of attendance, it may also capture strong preferences for/against the invasiveness of SAVR. Furthermore, all participants interpreted their response to the practice questions correctly, and only a small proportion of respondents demonstrated low health literacy or numeracy. This provides some reassurance that the preference heterogeneity observed in this study reflects a genuine difference in preference, rather than being the result of patients failing to complete the survey in a meaningful way. 

Two other studies that used ASW to elicit patient preferences have been published ^26, 27^. Both studies also observed substantial preference heterogeneity. One of these studies ^27^ provided evidence supporting the validity and reliability of the preference outputs, both by replicating the results of the ASW with a thresholding exercise, and by comparing participants’ responses with their qualitative statements on the basis for their answers. This provides some reassurance about the validity of responses to the ASW exercise used for the current study. This may suggest that methods such as ASW, which elicit individual-level patient preferences, capture more preference heterogeneity than population-level methods, such as discrete choice experiments. Further work could usefully continue to validate the results of ASW exercises and test the hypothesis that individual-level preferences method captures greater heterogeneity in patient preferences.

Only one other study of AS patients’ treatment preferences has been published to date ^28^. The study design was sufficiently different to the current study—focusing on patients’ willingness to accept the mortality risk associated with interventions—that it is not possible to directly compare the results. However, the study by Hussain *et al*. ^28^ did reveal a higher risk tolerance among patients with greater disease burden (defined as weekly incidence of restricting symptoms, perceived change in health compared with 1 year earlier, EQ-VAS scores, and the New York Heart Association (NYHA) classification). Our study failed to identify an association between patient preferences and NYHA classification, though this might be due to the limited sample size and the small proportion of the sample in the more severe stages of the NYHA classification. Further research could usefully gather data from a larger sample of AS patients to determine the association of preferences and patient characteristics, such as NYHA classification or whether patients have previously undergone treatment for AS.

While a majority of patients in the current study preferred TAVR, a number of patients (around 20%) preferred SAVR. This, and the underlying heterogeneity in patient preferences, support the need for a shared decision-making (SDM) tool that will help patients and surgeons choose procedures based on both clinical indications and patient risk tolerance. The Patient-Centered Outcomes Research Institute (PCORI) has developed a SDM tool to support patients choose between SAVR and TAVR ^15^. However, this tool includes a narrower range of treatment attributes—stroke risk, mortality risk and discharge home—than those included in the analysis reported in this study. Furthermore, the tool does not include a component to elicit a patient’s preferences.

As always, the conclusions of the study should be drawn in light of its limitations. First, it was not possible to engage patients in the design of the elicitation exercise. Instead, the patient voice was reflected in the design through a review of the limited preference research undertaken with AS patients to date and through the engagement that the experts who were consulted had themselves had with patients. Further work would usefully confirm with patients that no attributes were excluded from the study. Second, in the first survey round we relied on patient self-report of their AS diagnoses and severity, and in the second wave providing confirmation of AS diagnosis was voluntary. Third, the sample is healthier and younger than the population currently eligible for TAVR and SAVR ^29^. AS patients were recruited from the membership of Heart Valve Voice and Mended Hearts as well as through M3 Global Research, and it is possible that patients who are motivated to join these organizations may have different preferences than the broader population. Further AS patient preference research should replicate this study, including older patients with more severe disease burden. Fourth, it was necessary to assume that partial value functions were linear. The sample size and the number of attributes for which preferences were being collected meant collecting data on the shape of the partial value functions would have overburdened respondents.

Finally, the application of ASW in BRA is relatively novel and raises a number of questions. First, it relies on the idea that patients who are indifferent between adding one or another feature to a good or service would be willing to accept either improvement. The concept of indifference is commonly evoked in methods to assess the value of changes in health outcome. Further, the baseline against which improvements are assessed is the worse level of performance of the treatments being evaluated and over 80% of patients had previously been treated for AS. Thus, in most cases it might be reasonable to assume that patients would accept the changes, as they represent outcomes better than those they’ve already accepted. However, to infer that they would be willing to accept improvements in outcomes assumes that they were well informed when they made these treatment choices. Further research should test participants willingness to accept changes in attributes.

Second, unlike some other elicitation methods, such as discrete choice experiments, the study assumes that respondents don’t make mistakes when they are giving their answers, which is unlikely if respondents are near to their indifference points. It is thus necessary to assume that any such mistakes average out when assessing preferences for the study sample.

Third, as all respondents start their evaluation of attributes at the same point, ASW may be subject to anchoring. To mitigate this risk, ‘worst’ levels in the choice tasks were defined as the ‘worst’ clinically relevant level. However, in some instances this may have introduced a ceiling effect, with insufficient improvement in the attribute available to identify the indifference point. In these instances, it was necessary to increase the ‘worst’ level beyond that experienced by patients. Further work could usefully explore this apparent tension between anchoring and ceiling effects inherent within ASW.

## Conclusions

Most AS patients are willing to tolerate sizable increases in clinical risk in exchange for the benefits associated with TAVR. A BRA incorporating data from patients’ preferences for the attributes of AS treatments revealed a strong preference for TAVR compared to SAVR. The analysis also revealed substantial heterogeneity in individual patient preferences, partly associated with patient age. Further work is required to understand this heterogeneity, and whether additional patient characteristics such as NYHA class are associated with different preferences. In the meantime, SDM tools should incorporate the factors identified in this model to assist patients and clinicians in achieving a more patient-centered treatment decision.

## Data availability

### Underlying data

Open Science Framework: AS patient preference data. DOI 10.17605/OSF.IO/UGD8X ^24^.

This project contains the following underlying data:

- TAVR Manuscript_Full Dataset_Updated.xlsx (full dataset of patient responses including codebook).

### Extended data

Open Science Framework: AS patient preference data. DOI 10.17605/OSF.IO/UGD8X ^24^.

This project contains the following extended data:

- TAVR Manuscript Appendices S1_S2_Updated.docx (contains S1 Appendix, including S1 Table 1 and legends for S1 Figures 1-6; and S2 Appendix, including legends for S2 Figures 1-6).
- TAVR Manuscript Figures_Updated.docx (S1 Figures 1-6 and S2 Figures 1-6).
- TAVR Survey Contents_Updated.docx (a copy of the questionnaire given to each participant).

Data are available under the terms of the Creative Commons Zero “No rights reserved” data waiver (CC0 1.0 Public domain dedication).
