# Supplementary material for: Patient-centered benefit-risk analysis of transcatheter aortic valve replacement
Source: F1000Res. 2021 Feb 8;8:394. Originally published 2019 Apr 8. [Version 5] doi: 10.12688/f1000research.18796.5 (PMC6544076; doi:10.12688/f1000research.18796.5)
Supplement: Peer review history

# Peer review: Reviewer response for version 5

**DOI:** 10.5256/f1000research.54334.r79093

**Published:** 2021-02-16

**Authors:** Juan Marcos Gonzalez

**Affiliations:** 1 Department of Medicine, Duke University School of Medicine, Durham, NC, USA

I thank the authors for addressing my previous concerns. The changes and clarifications offered have addressed my comments satisfactorily.

 My only remaining comment is in response to the authors’ question about the potential insufficiency of equivalences to measure unbiased marginal rates of substitution. There is certainly some literature in DCEs showing that inappropriate use of a forced-choice format can lead to variation in parameter estimates (Determann et al., 2019 ^1^; and Campbell and Erdem, 2019 ^2^). While the need for an opt-out or status-quo alternative depends on the study objectives and the relevant decision context, there is also evidence that marginal rates of substitution change with the inclusion of cost in a forced-choice format (Pedersen et al., 2011 ^3^).

Is the work clearly and accurately presented and does it cite the current literature?

Partly

If applicable, is the statistical analysis and its interpretation appropriate?

Partly

Are all the source data underlying the results available to ensure full reproducibility?

Partly

Is the study design appropriate and is the work technically sound?

Partly

Are the conclusions drawn adequately supported by the results?

Partly

Are sufficient details of methods and analysis provided to allow replication by others?

Yes

Reviewer Expertise:

NA

I confirm that I have read this submission and believe that I have an appropriate level of expertise to confirm that it is of an acceptable scientific standard.

## References

[1] : Designing Unforced Choice Experiments to Inform Health Care Decision Making: Implications of Using Opt-Out, Neither, or Status Quo Alternatives in Discrete Choice Experiments. *Med Decis Making*.39(6) : 10.1177/0272989X19862275 681-692 10.1177/0272989X19862275 31354031

[2] : Including Opt-Out Options in Discrete Choice Experiments: Issues to Consider. *Patient*.12(1) : 10.1007/s40271-018-0324-6 1-14 10.1007/s40271-018-0324-6 30073482

[3] : Does the Inclusion of a Cost Attribute in Forced and Unforced Choices Matter?. *Journal of Choice Modelling*.2011;4(3) : 10.1016/S1755-5345(13)70044-7 88-109 10.1016/S1755-5345(13)70044-7

# Peer review: Reviewer response for version 4

**DOI:** 10.5256/f1000research.25257.r63117

**Published:** 2020-11-02

**Authors:** Juan Marcos Gonzalez

**Affiliations:** 1 Department of Medicine, Duke University School of Medicine, Durham, NC, USA

Thank you for the opportunity to review this interesting manuscript. I commend the authors for looking at such a novel method for the elicitation of risk tolerance with stated-preference methods. The manuscript is well-written and the explanation of the method is clear. I do have some major concerns that I think should be addressed by the authors. I summarized my concerns below.

 My biggest concern is the number of assumptions implicit in the derivation of the MAR and MAB measures with these data. In particular, the operating assumption that value equivalence in the context of the decisions elicited implies acceptability. I would agree with that premise if the question presented the right context, but here things seem a bit murky. Does the fact that I am indifferent between adding any one of two features to a good or service imply that I'm willing to accept either improvement? Not necessarily. While this assumption is not necessarily wrong, I think it needs to be better justified either with evidence from their qualitative/pilot work or through previous literature.

 Almost equally problematic is the omission of measurement error in the analysis of these data. Respondents answered three questions with each pairwise comparison. Depending on the step procedure to change the specifics of the comparisons, there could be a systematic error structure for the measures. For example, a 1-unit improvement across questions could systematically fall short of a threshold among respondents who truly require low probabilities of adverse events to achieve equivalence. Conversely, an initial full swing in the relevant range followed by a gradual increase in the risk levels could end up overestimating the equivalences. I could not find any details on the algorithm followed by the authors as they tried to identify an indifference threshold with only three questions per comparison. That information needs to be included or highlighted.

 Even if the respondent reached an equivalence threshold with the questions in the survey, there is still a measurement error for at least three reasons:

1. The current analysis assumes that responses are collected without errors and that respondents don’t make mistakes as they give their answers. This is particularly dubious when respondents are near indifference and the attributes are relatively important (i.e., the equivalence ratio is small).
2. The study design ignores anchoring problems as all respondents started their evaluation of attribute equivalence at the same point. It is well known that anchoring problems can bias measures of preferences. Potentially, this would not be problematic if the baseline scenario was clinically relevant—like the current standard of care—but the principle for the baseline levels seems to be unrelated to clinical considerations.
3. The range established a priori for the swings may not be sufficient to achieve indifference for some attributes. This would be considered a ceiling effect and could overestimate the importance weight of some attributes.

Without some sense of the measurement error of the instrument, either through theory (i.e., random utility theory) or empirical data (i.e., as done with PROMs), we would seem to be blind about the validity of the study results. These measurement issues need to be better addressed or included as limitations of the study.

 Another important implicit assumption of the method is that of linearity or proportionality of weights within the attribute range. There is a lot of evidence that this assumption often does not hold, particularly with low risks of events. The assumption would seem to be particularly crucial here because the evaluation of the importance is not done in the direction (and potentially the range) of the relevant risk changes from a regulatory standpoint. That is, the authors evaluate the impact of reducing the risks from a high absolute level and then move the approximated equivalences to a different point within the studied range to predict the importance of increases in risk levels.

 If data comes from the same clinically relevant range the authors could at least argue that the average effects within the range are recoverable, but if evaluating a completely different risk range, there is no guarantee that the linearity assumption is adequate even for the average effect.

 Consider the following *extreme *example, suppose we were interested in the importance of increasing the risk of death within the range of 0% to 10%. Collecting preference information over that range, even if we imposed linearity or proportionality of preferences, would at least capture the correct average effect within that increase—assuming we use the right analysis tools. However, if instead, we approximate that information by looking at the importance of reducing the risk of death from 100% to 90%, there is no guarantee that this importance would be an accurate approximation of preferences for 0% to 10%. The only way the two measures would be identical is if the linearity assumption imposed by the authors holds for the full range of risk levels, but the authors don’t test this to justify such an important—and potentially flawed—assumption.

 Overall, I think this study results could offer a reasonable approximation of risk tolerance with adaptive swing weighting, but the validity of such approximation is unclear without further evaluation of the implicit assumptions made to derive MAR and MAB.

Is the work clearly and accurately presented and does it cite the current literature?

Partly

If applicable, is the statistical analysis and its interpretation appropriate?

Partly

Are all the source data underlying the results available to ensure full reproducibility?

Partly

Is the study design appropriate and is the work technically sound?

Partly

Are the conclusions drawn adequately supported by the results?

Partly

Are sufficient details of methods and analysis provided to allow replication by others?

Yes

Reviewer Expertise:

Health preference research and the use of these preference measures to infer risk tolerance.

I confirm that I have read this submission and believe that I have an appropriate level of expertise to state that I do not consider it to be of an acceptable scientific standard, for reasons outlined above.

# Response

**Published:** 2021-01-29

**Authors:** Kevin Marsh

**Affiliations:** Evidera, UK

Reviewer Feedback 1

 My biggest concern is the number of assumptions implicit in the derivation of the MAR and MAB measures with these data. In particular, the operating assumption that value equivalence in the context of the decisions elicited implies acceptability. I would agree with that premise if the question presented the right context, but here things seem a bit murky. Does the fact that I am indifferent between adding any one of two features to a good or service imply that I'm willing to accept either improvement? Not necessarily. While this assumption is not necessarily wrong, I think it needs to be better justified either with evidence from their qualitative/pilot work or through previous literature.

*Authors' Response 1*

*Thanks for this observation. This is an interesting question. Given the centrality of the concept of indifference in defining utility in economic theory, the associated notion of the marginal rate of substitution, and employment of the concept of indifference in various other preference elicitation methods – not least TTO, we had not questioned the relationship between indifference and willingness to accept. We have reference the above in justifying the approach. Though we’d be interested in the reviewers thoughts on whether there is a literature which challenges this notion that we are not familiar with.*

Reviewer Feedback 2

 Almost equally problematic is the omission of measurement error in the analysis of these data. Respondents answered three questions with each pairwise comparison. Depending on the step procedure to change the specifics of the comparisons, there could be a systematic error structure for the measures. For example, a 1-unit improvement across questions could systematically fall short of a threshold among respondents who truly require low probabilities of adverse events to achieve equivalence. Conversely, an initial full swing in the relevant range followed by a gradual increase in the risk levels could end up overestimating the equivalences. I could not find any details on the algorithm followed by the authors as they tried to identify an indifference threshold with only three questions per comparison. That information needs to be included or highlighted.

*Authors' Response 2*

**

*The algorithm used is reported in Extended Data, Appendix 1. We did not adopt either of the approaches that cause the reviewer some concern. Rather, each iteration of the level of performance cut the remaining space where the indifferent point was located in half.*

Reviewer Feedback 3

 Potential measurement error because: The current analysis assumes that responses are collected without errors and that respondents don’t make mistakes as they give their answers. This is particularly dubious when respondents are near indifference and the attributes are relatively important (i.e., the equivalence ratio is small).

*Authors' Response 3*

**

*We agree this is a key difference between swing weighting and methods like discrete choice experiments. We have added a note to this effect in the paragraph on limitations in the discussion section.*

Reviewer Feedback 4

 Potential measurement error because: The study design ignores anchoring problems as all respondents started their evaluation of attribute equivalence at the same point. It is well known that anchoring problems can bias measures of preferences. Potentially, this would not be problematic if the baseline scenario was clinically relevant—like the current standard of care—but the principle for the baseline levels seems to be unrelated to clinical considerations.

*Authors' Response 4*

**

*We agree with the reviewers observations about the importance of anchoring. Where possible the ‘worst’ level in the choice tasks were defined as the worst level in the procedure choice to which the preference estimates were applied. However, in some instance the pilot suggested that this would not give us enough ‘runway’ to identify indifference – per the following comments, we risked ceiling effects if we did this. In those cases, the ‘worst’ level needed to be increased compared with that experienced by patients to create the ‘runway’. We agree this might be a source of bias and have added a note to this effect in the discussion section.*

Reviewer Feedback 5

 Potential measurement error because: The range established a priori for the swings may not be sufficient to achieve indifference for some attributes. This would be considered a ceiling effect and could overestimate the importance weight of some attributes

*Authors' Response 5*

**

*As noted in response to the previous comment, the range over which swings were explored was sometimes widened, precisely to try to avoid such ceiling effects. Nevertheless, the bi-modal distribution observed points to the potential that such effects may not have been entirely eradicated. Also noted above, in such designs it is possible that there is a tension between efforts to avoid anchoring and efforts to avoid ceiling effects. We have added a note on this in the discussion section.*

Reviewer Feedback 6

 Another important implicit assumption of the method is that of linearity or proportionality of weights within the attribute range. There is a lot of evidence that this assumption often does not hold, particularly with low risks of events. The assumption would seem to be particularly crucial here because the evaluation of the importance is not done in the direction (and potentially the range) of the relevant risk changes from a regulatory standpoint. That is, the authors evaluate the impact of reducing the risks from a high absolute level and then move the approximated equivalences to a different point within the studied range to predict the importance of increases in risk levels. If data comes from the same clinically relevant range the authors could at least argue that the average effects within the range are recoverable, but if evaluating a completely different risk range, there is no guarantee that the linearity assumption is adequate even for the average effect.

*Authors' Response 6*

**

*We agree with this limitation. We had included this in the discussion of the limitations of the approach. We’ve re-emphasized this challenge in the discussion section, and call it out as a potential limit of the ASW method when working with more than a few attributes. The method was adopted as the goal of the study was to collect of data on preferences for many attributes with small sample sizes. However, in this context, it was not possible to also test the non-linearity of value functions without overburdening participants.*

# Peer review: Reviewer response for version 2

**DOI:** 10.5256/f1000research.21135.r48437

**Published:** 2019-05-29

**Authors:** Ross Jaffe

**Affiliations:** 1 Versant Ventures, San Francisco, CA, USA

The authors have addressed my major concerns, and I support indexing of this article.

Is the work clearly and accurately presented and does it cite the current literature?

Yes

If applicable, is the statistical analysis and its interpretation appropriate?

I cannot comment. A qualified statistician is required.

Are all the source data underlying the results available to ensure full reproducibility?

Yes

Is the study design appropriate and is the work technically sound?

Yes

Are the conclusions drawn adequately supported by the results?

Yes

Are sufficient details of methods and analysis provided to allow replication by others?

Yes

Reviewer Expertise:

Internal medicine physician with prior experience in outcomes research and shared decision making. Not actively involved in research in my present role as a venture capitalist, but I led the MDIC Patient Centered Benefit-Risk Project that developed a framework for incorporating patient preferences into FDA medical device regulation and developed the first catalog of patient preference methodologies included as an appendix to that report.

I confirm that I have read this submission and believe that I have an appropriate level of expertise to confirm that it is of an acceptable scientific standard.

# Peer review: Reviewer response for version 2

**DOI:** 10.5256/f1000research.21135.r48436

**Published:** 2019-05-21

**Authors:** Megan Coylewright

**Affiliations:** 1 Dartmouth-Hitchcock Medical Center, Lebanon, NH, USA

The changes provided needed updates. No major changes to methods or conclusions.

Is the work clearly and accurately presented and does it cite the current literature?

Yes

If applicable, is the statistical analysis and its interpretation appropriate?

I cannot comment. A qualified statistician is required.

Are all the source data underlying the results available to ensure full reproducibility?

Partly

Is the study design appropriate and is the work technically sound?

Yes

Are the conclusions drawn adequately supported by the results?

Partly

Are sufficient details of methods and analysis provided to allow replication by others?

Yes

Reviewer Expertise:

shared decision making

I confirm that I have read this submission and believe that I have an appropriate level of expertise to confirm that it is of an acceptable scientific standard.

# Peer review: Reviewer response for version 1

**DOI:** 10.5256/f1000research.20591.r46834

**Published:** 2019-05-07

**Authors:** Megan Coylewright

**Affiliations:** 1 Dartmouth-Hitchcock Medical Center, Lebanon, NH, USA

The co-authors have an ambitious agenda to identify patient preferences regarding the differing attributes for the treatment of aortic stenosis. They are to be commended for tackling an important topic, and employing known methodologic standards for addressing the question.

 They deploy a methodology of adapted swing weighting (ASW), identifying which treatment attributes patients would tolerate to move from an invasive (surgical aortic valve replacement, or SAVR) option to a minimally invasive (transcatheter aortic valve replacement, or TAVR) option. A set of pairwise comparisons were made between 'less invasive" and other features of AVR, with 5 different iterations presented.

 The study is written by employees of one of the TAVR companies (Edwards LifeSciences), as well as employees of a company that specializes in patient preference work, paid for by Edwards. It is not apparent that there was engagement of other stakeholders in the study design, such as patients, families, clinicians, administrators, policy makers, etc. There were 5 patients with the disease condition that trialed the exercises before the start of the study to ensure understandability. There is no information how these patients were recruited, or what changes were made in response to their feedback.

 The authors’ conclusions are that patients are willing to tolerate big increases in risk and exchange for benefits of a minimally invasive procedure.

 Importance of the research question: Given the recent reconsideration of the TAVR national coverage determination, data as needed to inform interventions designed to increase the degree to which patient preferences are included in final decision making. This study suggests that there is heterogeneity in patient preferences and that patients may tolerate larger differences in risk than physicians and regulators traditionally have deemed acceptable when faced with a minimally invasive procedure.

 Originality of the work: There is not a large amount of data on the preferences of patients for TAVR. 

 Strengths:

 Identifying patient preferences and how they influence decision-making is an important topic.  Pair-wise comparisons can be helpful to delineate tolerance of risk in one domain versus another.

 In the decision aid that was presented to the patient's demonstrating differences between the 2 therapies, the same picture was used for both surgery and transcatheter procedure which did potentially reduce bias.

 Weaknesses:

 The largest weakness is the fact that the attributes were not selected by patients known to be affected by the disease condition; a survey was asked of participants in advocacy groups online.  The eligible patients were created from the membership of heart valve voice and mended hearts.  They were directed to an online screening tool where patient reported disease conditions were recorded. The study sample was largely white and highly educated. No patients had severe limitations to physical activity, which has been shown to significantly impact decision-making.  This has been a consistent weakness in study design for clinical trials as well. The patients selected for the survey, in addition, were not necessarily patients that would be considering this decision.

 Data analysis:

 Responses from the survey were used to compute the maximum acceptable increase in risk or maximum acceptable reduction in benefits that participants would be willing to tolerate in exchange for being involved in a less invasive procedure.

 Interpretation of data:

 93 patients completed the survey. Patients were not representative of the TAVR population and described above in the weaknesses. The authors importantly describe substantial heterogeneity and patient's responses to the exercise. Importantly, this correlated with patient age, which was lower in the sample than the TAVR population. Patients were more likely to prefer TAVR when considering benefits of independence or risks of mortality or disabling stroke. They were more likely to favor surgical AVR when considering risks of pacemaker and uncertainty regarding durability.

 Presentation of data and story:

             Abstract: The abstract describes that the purpose of the research is to determine which outcomes associated with TAVR and S AVR patients consider most important. Patients were not included in the selection of potential outcomes associated with the therapies. The abstract is stand-alone, describing the findings of trade-offs for risks and benefits. However, there should be a further statement about whether or not the patients had aortic stenosis and whether they were symptomatic as this is known to impact decision-making.

             Introduction: The introduction would need to be revised regarding the literature around pacemaker and stroke. Otherwise, the introduction lays out the rationale and explains the goals for the study.

             Methods: The methods provide sufficient detail that experiments could be repeated however, much of the methods could be relegated to a supplement online.

             Results: The results are hypothesis generating but are limited in the reliability given uncertainty regarding patient diagnosis, severity of AS and symptoms status. In addition, the attributes that the patients are commenting on are defined by the research team, with leadership from the company, and therefore have potential bias. 

             The findings are clinically relevant as they describe differences and patient preferences around risk and benefit. They highlight the need for shared decision making approach. They also raise the issue of which outcomes are most important to patients. Additional research is needed to identify that from patients with symptoms themselves.

             Discussion: The discussion around the relationship between the guidelines and regulators needs editing. It is not clear on what the authors mean when they say that the current guidelines "do not take into account recent clinical data supporting the use of TAVR". There is an important point about how patients willingness to tolerate certain risks may differ from policy makers and physicians and an argument can be made to include patient preferences into final decision making.

 Limitations described include how well the participants responding to the survey paid attention to the questions. This is described as a reason for some of the heterogeneity. Limitation would be the small size of the patient's sample and those patients not having the disease that is being studied nor being symptomatic. The limitations focus primarily on the selection of ASW exercises as a way to assess patient preferences rather than the patient population.

             The tables and figures do stand-alone. The references appear appropriate.

 I appreciate the opportunity to review this interesting manuscript.

Is the work clearly and accurately presented and does it cite the current literature?

Yes

If applicable, is the statistical analysis and its interpretation appropriate?

I cannot comment. A qualified statistician is required.

Are all the source data underlying the results available to ensure full reproducibility?

Partly

Is the study design appropriate and is the work technically sound?

Yes

Are the conclusions drawn adequately supported by the results?

Partly

Are sufficient details of methods and analysis provided to allow replication by others?

Yes

Reviewer Expertise:

shared decision making

I confirm that I have read this submission and believe that I have an appropriate level of expertise to confirm that it is of an acceptable scientific standard, however I have significant reservations, as outlined above.

# Response

**Published:** 2019-05-08

**Authors:** Kevin Marsh

**Affiliations:** Evidera, UK

**Review 2: Meghan Coylewright**

 The co-authors have an ambitious agenda to identify patient preferences regarding the differing attributes for the treatment of aortic stenosis. They are to be commended for tackling an important topic, and employing known methodologic standards for addressing the question.

 They deploy a methodology of adapted swing weighting (ASW), identifying which treatment attributes patients would tolerate to move from an invasive (surgical aortic valve replacement, or SAVR) option to a minimally invasive (transcatheter aortic valve replacement, or TAVR) option. A set of pairwise comparisons were made between 'less invasive" and other features of AVR, with 5 different iterations presented.

 The study is written by employees of one of the TAVR companies (Edwards LifeSciences), as well as employees of a company that specializes in patient preference work, paid for by Edwards. It is not apparent that there was engagement of other stakeholders in the study design, such as patients, families, clinicians, administrators, policy makers, etc. There were 5 patients with the disease condition that trialed the exercises before the start of the study to ensure understandability. There is no information how these patients were recruited, or what changes were made in response to their feedback.

 The authors’ conclusions are that patients are willing to tolerate big increases in risk and exchange for benefits of a minimally invasive procedure.

 Importance of the research question: Given the recent reconsideration of the TAVR national coverage determination, data as needed to inform interventions designed to increase the degree to which patient preferences are included in final decision making. This study suggests that there is heterogeneity in patient preferences and that patients may tolerate larger differences in risk than physicians and regulators traditionally have deemed acceptable when faced with a minimally invasive procedure.

 Originality of the work: There is not a large amount of data on the preferences of patients for TAVR. 

 Strengths:

 Identifying patient preferences and how they influence decision-making is an important topic.  Pair-wise comparisons can be helpful to delineate tolerance of risk in one domain versus another.

 In the decision aid that was presented to the patient's demonstrating differences between the 2 therapies, the same picture was used for both surgery and transcatheter procedure which did potentially reduce bias.

 Weaknesses:

 The largest weakness is the fact that the attributes were not selected by patients known to be affected by the disease condition; a survey was asked of participants in advocacy groups online.  The eligible patients were created from the membership of heart valve voice and mended hearts.  They were directed to an online screening tool where patient reported disease conditions were recorded. The study sample was largely white and highly educated. No patients had severe limitations to physical activity, which has been shown to significantly impact decision-making.  This has been a consistent weakness in study design for clinical trials as well. The patients selected for the survey, in addition, were not necessarily patients that would be considering this decision.

***Response: We thank the reviewer for their interest in the study and their comments. We agree with concerns about the representative of the sample. The observation of the limitation of the study has been extended in the discussion section to read:***

***“Finally, the current study is based on a relatively small sample of patients, and the sample is healthier and younger than the population currently eligible for TAVR and SAVR. AS patients were recruited from the membership of Heart Value Voice and Mended Hearts, and it is possible that patients who are motivated to join these organizations may have different preferences than the broader population. Further AS patient preference research should replicate this study in a larger sample of patients, including more patients with more severe disease burden”.***

 Data analysis:

 Responses from the survey were used to compute the maximum acceptable increase in risk or maximum acceptable reduction in benefits that participants would be willing to tolerate in exchange for being involved in a less invasive procedure.

 Interpretation of data:

 93 patients completed the survey. Patients were not representative of the TAVR population and described above in the weaknesses. The authors importantly describe substantial heterogeneity and patient's responses to the exercise. Importantly, this correlated with patient age, which was lower in the sample than the TAVR population. Patients were more likely to prefer TAVR when considering benefits of independence or risks of mortality or disabling stroke. They were more likely to favor surgical AVR when considering risks of pacemaker and uncertainty regarding durability.

 Presentation of data and story:

             Abstract: The abstract describes that the purpose of the research is to determine which outcomes associated with TAVR and S AVR patients consider most important. Patients were not included in the selection of potential outcomes associated with the therapies. The abstract is stand-alone, describing the findings of trade-offs for risks and benefits. However, there should be a further statement about whether or not the patients had aortic stenosis and whether they were symptomatic as this is known to impact decision-making.

***Response: The following statement has been added to the abstract: All patients self-reported as being diagnosed with AS, and as either having received treatment for their AS in the previous 10 years or as experiencing limitations in their physical activity as a result of their AS.***

             Introduction: The introduction would need to be revised regarding the literature around pacemaker and stroke. Otherwise, the introduction lays out the rationale and explains the goals for the study.

***Response: We have not updated the introduction. Perhaps the reviewer could provide more detail on the literature that should be summarized in the introduction. *** 

             Methods: The methods provide sufficient detail that experiments could be repeated however, much of the methods could be relegated to a supplement online.

***Response: We have not moved any content to a supplementary appendix. We would be happy to consider doing so if the editors thought that was more appropriate given the audience for the journal.***

             Results: The results are hypothesis generating but are limited in the reliability given uncertainty regarding patient diagnosis, severity of AS and symptoms status. In addition, the attributes that the patients are commenting on are defined by the research team, with leadership from the company, and therefore have potential bias. 

***Response: We agree that it would have been preferable to have been able to engage patients more in the design of the study. The following statement has been added to the discussion section:***

***“it was not possible to engage patients in the design of the elicitation exercise. Instead, the patient voice was reflected in the design through a review of the limited preference research undertaken with AS patients to date and through the engagement that the experts who were consulted had themselves had with patients. Further work would usefully confirm with patients that no attributes were excluded from the study”.***

***We have also added to the paragraph in the discussion on limitations the fact that we relied on patient self report of their diagnoses and AS severity.***

             The findings are clinically relevant as they describe differences and patient preferences around risk and benefit. They highlight the need for shared decision making approach. They also raise the issue of which outcomes are most important to patients. Additional research is needed to identify that from patients with symptoms themselves.

***Response: We agree this would improve the research. As above, a note to this effect has been added to the discussion.***

             Discussion: The discussion around the relationship between the guidelines and regulators needs editing. It is not clear on what the authors mean when they say that the current guidelines "do not take into account recent clinical data supporting the use of TAVR". There is an important point about how patients willingness to tolerate certain risks may differ from policy makers and physicians and an argument can be made to include patient preferences into final decision making.

 Limitations described include how well the participants responding to the survey paid attention to the questions. This is described as a reason for some of the heterogeneity. Limitation would be the small size of the patient's sample and those patients not having the disease that is being studied nor being symptomatic. The limitations focus primarily on the selection of ASW exercises as a way to assess patient preferences rather than the patient population.

***Response: See earlier responses. The discussion of the limitations of the study has been extended to cover these concerns.***

# Peer review: Reviewer response for version 1

**DOI:** 10.5256/f1000research.20591.r46833

**Published:** 2019-04-25

**Authors:** Ross Jaffe

**Affiliations:** 1 Versant Ventures, San Francisco, CA, USA

This article by Marsh et al assesses patient preferences regarding the important medical decision about whether to undergo traditional surgical aortic valve replacement (SAVR) versus the newer transcatheter aortic valve replacement (TAVR) for aortic stenosis. This is an important effort to understand what attributes of benefit and risk from aortic valve replacement are most important to patients and understand how patients tradeoff those attributes in deciding which therapy to choose.  With shared decision-making becoming increasingly used as our US healthcare system evolves to be more patient-centric, studies such as this one are important to expanding our understanding of how best to provide the information needed by patients to make informed decisions about their care.  This study confirms a belief that many clinicians have that most patients prefer TAVR to SAVR because it is less invasive, providing evidence that about 75% patients prefer the less invasive approach.  These patients are willing to tolerate a somewhat higher risk of stroke, pacemaker placement, and dialysis, and less evidence of long-term duration for the benefits of less invasiveness.  It also identifies that younger patients (<60 yo) perspectives may differ from those of older patients (>60 yo) somewhat, although both groups generally prefer the less invasive procedure. 

 I support the publication of this study.  While this study is not perfect from my point of view, it is an important contribution to the literature, both about how to treat aortic stenosis and about patient preference assessment more generally.  Understanding what treatment attributes are most important to patients and how patients trade off benefits and risks is important to understanding how best to inform patients about their treatment options and help them make decisions that best reflect their individual preferences.  As the paper notes on p. 11, there has only been one other, more limited study of patient preferences in aortic stenosis, so this study is an important expansion of our understanding of patient preferences that clinical area.  

 Additionally, from a methodological perspective and as also noted in the paper, there are only a few other studies that use adapted swing weights (ASW) to assess patient preferences.  Swing weighting is one of only fourteen methods identified in the Medical Device Innovation Consortium (MDIC) review of preference assessment methodologies.  (See: MDIC Patient Centered Benefit Risk Project Report, Appendix A: Catalog of Methods for Assessing Patient Preferences for Benefits and Harms of Medical Technologies, May 2015, available at: https://mdic.org/resource/patient-centered-benefit-risk-pcbr-framework/).  This study is an important contribution to the literature about swing weighting methods, and should allow comparison to studies of patient preference using other methods to help researchers and clinicians better understand how best to assess patient preferences.

 This study does have a few issues that should be highlighted to help put the results in context.  I also note an area for further assessment of the data as well as areas for future research focus.  Please note that I come at this study as someone with an interest in patient preferences from clinical and regulatory policy perspective, and do not have the expertise to comment on the specific methodology or statistical analysis, which I will leave to experts in those areas.

 Major concerns:

 1. The mixing of treated and untreated patients in the participant population:  In Box 1 on p. 6, the inclusion criteria describe that patients in the study could have already had a procedure within the last 10 years or could be untreated.  From a shared decision making point of view, patients express their preferences prior to treatment – so it is most important to understand the benefit-risk attributes that are most important to patients not yet treated and how such patients trade off such risks.  The mixing of both untreated and treated patients may make it hard to understand how the pre-procedure patients view these issues. 

 Prior treatment could significantly influence a patient’s preference for one treatment option or another, but it his hard to know *a priori *how treatment would influence a patients preferences.  Prior treatment may introduce a confirmation bias that patients tend to prefer the procedure that they chose to have, and therefore require much greater benefit or much less risk from the alternative procedure compared to treatment naïve patients.  Alternatively, if patients had a negative experience with their prior procedure, they may find the benefit/risk profile of the alternative procedure much more attractive than that of the procedure that they had.  Additionally, their experience of specific benefits or risks from their procedure may skew their weighting of those specific attributes compared to other patients.

 For this study, it would be important in Table 4 to add a breakdown of the patient treatment history, specifically the number and percentage of patients that have had SAVR, TAVR, or were untreated.  

 Additionally, it would be helpful to add a comparison of the preferences of patients in each of these categories to show how prior treatment affects the MIR/MRB for each attribute, perhaps in a table similar to Table 5 except substituting treatment category for age.  One concern is that with a sample size of 93 patients, sub-categorization by treatment status may result in too few patients in any one category to have confidence in the results.  If this is a problem, it should be acknowledged that future studies may be needed to better understand the effect of prior treatment on preferences in aortic valve replacement.

 2. Representativeness of patients involved in member organizations:  The study recruited participants from two member organizations (Heart Value Voice and Mended Hearts).  While it is understandable why the membership of these organizations facilitated identifying patients with aortic valve disease. However, patients who are motivated enough to join such organizations may have different preferences than the broader population of patients with the disease eligible for treatment of their aortic valve disease.  There is no way to assess this potential difference in this study, but the authors could acknowledge this potential source of bias in the sample population in their discussion of the results and encourage future study in one or more different aortic valve disease populations. 

 3. Lack of clarity in the attribute of “Time over which the procedure has been proven to work”:  From the definition of this attribute in the paper, it is difficult to know whether patients interpreted this attribute as a measure of how long they could expect benefit, i.e, duration of effect, or whether patients also viewed this as how much clinical experience there was with a treatment, i.e., uncertainty in the knowledge about the effect.  (See section 2 of the MDIC Patient Centered Benefit Risk Report for a nice discussion of uncertainty and how it relates to patient preferences.). This ambiguity raises the question about whether this attribute as described elicited preferences about expected duration, or elicited preferences about patient tolerance of uncertainty about the effect of TAVR, or some combination of the two.  Showing an example of how this “proven to work” attribute was shown to patients akin to Figure 1 might clarify this ambiguity.  Future studies might try to separate these attributes, particularly comparing an established therapy like SAVR with a newer treatment like TAVR. 

 Minor issues and typos:

1. Introduction, last paragraph of left column, p. 3:  the beginning of the second sentence is awkward: “However, little is known about the weights that patients assign to which attributes, . . . . “  I would suggest “However, little is known about the weights that patients assign to each attribute. . . .” 
2. Methods, Attribute selection, last sentence (p.3): note “was were”.  I would remove the were. 
3. Table 1, p. 4: Disabling non-fatal stroke:  in the description, first sentence, note the “one 1” – should be “one month”. 
4. Table 2, p. 4, description of “independence” attribute:  the description of the measure of this attribute does not mention that this is measured at one month, whereas all the other measures note the time frame of the measure.  
5. Analysis, p. 6, top right column, first paragraph: there looks to be a calculation error in the next to the last sentence.  4.8%/2% = 2.4, not 1.4 as written. 
6. Comparisons of TAVR and SAVR, p. 7: right column, top paragraph – “In each case, patents. . . . ” should be “In each case, patients. . . .”
7. Figures 2 and 3, p. 9:  Each x-axis should be labeled with what the unit of measurement is.  This will help the figures be more self-explanatory. 

I hope that these comments are helpful.  Given my interest in the use of patient preferences in the FDA regulatory process and increasing shared decision making in medicine broadly, I am pleased that the authors undertook this study to better understand patient preferences in aortic stenosis treatment, and I support its publication.  It should be a nice addition to both the aortic stenosis treatment literature and the patient preference literature.

Is the work clearly and accurately presented and does it cite the current literature?

Yes

If applicable, is the statistical analysis and its interpretation appropriate?

I cannot comment. A qualified statistician is required.

Are all the source data underlying the results available to ensure full reproducibility?

Yes

Is the study design appropriate and is the work technically sound?

Yes

Are the conclusions drawn adequately supported by the results?

Yes

Are sufficient details of methods and analysis provided to allow replication by others?

Yes

Reviewer Expertise:

Internal medicine physician with prior experience in outcomes research and shared decision making.  Not actively involved in research in my present role as a venture capitalist, but I led the MDIC Patient Centered Benefit-Risk Project that developed a framework for incorporating patient preferences into FDA medical device regulation and developed the first catalog of patient preference methodologies included as an appendix to that report.

I confirm that I have read this submission and believe that I have an appropriate level of expertise to confirm that it is of an acceptable scientific standard, however I have significant reservations, as outlined above.

# Response

**Published:** 2019-05-08

**Authors:** Kevin Marsh

**Affiliations:** Evidera, UK

**Review 1: Ross Jaffe**

This article by Marsh et al assesses patient preferences regarding the important medical decision about whether to undergo traditional surgical aortic valve replacement (SAVR) versus the newer transcatheter aortic valve replacement (TAVR) for aortic stenosis. This is an important effort to understand what attributes of benefit and risk from aortic valve replacement are most important to patients and understand how patients tradeoff those attributes in deciding which therapy to choose.  With shared decision-making becoming increasingly used as our US healthcare system evolves to be more patient-centric, studies such as this one are important to expanding our understanding of how best to provide the information needed by patients to make informed decisions about their care.  This study confirms a belief that many clinicians have that most patients prefer TAVR to SAVR because it is less invasive, providing evidence that about 75% patients prefer the less invasive approach.  These patients are willing to tolerate a somewhat higher risk of stroke, pacemaker placement, and dialysis, and less evidence of long-term duration for the benefits of less invasiveness.  It also identifies that younger patients (<60 yo) perspectives may differ from those of older patients (>60 yo) somewhat, although both groups generally prefer the less invasive procedure. 

I support the publication of this study.  While this study is not perfect from my point of view, it is an important contribution to the literature, both about how to treat aortic stenosis and about patient preference assessment more generally.  Understanding what treatment attributes are most important to patients and how patients trade off benefits and risks is important to understanding how best to inform patients about their treatment options and help them make decisions that best reflect their individual preferences.  As the paper notes on p. 11, there has only been one other, more limited study of patient preferences in aortic stenosis, so this study is an important expansion of our understanding of patient preferences that clinical area.  

Additionally, from a methodological perspective and as also noted in the paper, there are only a few other studies that use adapted swing weights (ASW) to assess patient preferences.  Swing weighting is one of only fourteen methods identified in the Medical Device Innovation Consortium (MDIC) review of preference assessment methodologies.  (See: MDIC Patient Centered Benefit Risk Project Report, Appendix A: Catalog of Methods for Assessing Patient Preferences for Benefits and Harms of Medical Technologies, May 2015, available at: https://mdic.org/resource/patient-centered-benefit-risk-pcbr-framework/).  This study is an important contribution to the literature about swing weighting methods, and should allow comparison to studies of patient preference using other methods to help researchers and clinicians better understand how best to assess patient preferences.

***Response: We thank the reviewer for their interest in our study***

This study does have a few issues that should be highlighted to help put the results in context.  I also note an area for further assessment of the data as well as areas for future research focus.  Please note that I come at this study as someone with an interest in patient preferences from clinical and regulatory policy perspective, and do not have the expertise to comment on the specific methodology or statistical analysis, which I will leave to experts in those areas.

Major concerns:

1. The mixing of treated and untreated patients in the participant population:  In Box 1 on p. 6, the inclusion criteria describe that patients in the study could have already had a procedure within the last 10 years or could be untreated.  From a shared decision making point of view, patients express their preferences prior to treatment – so it is most important to understand the benefit-risk attributes that are most important to patients not yet treated and how such patients trade off such risks.  The mixing of both untreated and treated patients may make it hard to understand how the pre-procedure patients view these issues. 

Prior treatment could significantly influence a patient’s preference for one treatment option or another, but it his hard to know *a priori *how treatment would influence a patients preferences.  Prior treatment may introduce a confirmation bias that patients tend to prefer the procedure that they chose to have, and therefore require much greater benefit or much less risk from the alternative procedure compared to treatment naïve patients.  Alternatively, if patients had a negative experience with their prior procedure, they may find the benefit/risk profile of the alternative procedure much more attractive than that of the procedure that they had.  Additionally, their experience of specific benefits or risks from their procedure may skew their weighting of those specific attributes compared to other patients.

For this study, it would be important in Table 4 to add a breakdown of the patient treatment history, specifically the number and percentage of patients that have had SAVR, TAVR, or were untreated.  

Additionally, it would be helpful to add a comparison of the preferences of patients in each of these categories to show how prior treatment affects the MIR/MRB for each attribute, perhaps in a table similar to Table 5 except substituting treatment category for age.  One concern is that with a sample size of 93 patients, sub-categorization by treatment status may result in too few patients in any one category to have confidence in the results.  If this is a problem, it should be acknowledged that future studies may be needed to better understand the effect of prior treatment on preferences in aortic valve replacement.

***Response: This is an important observation. We have updated Table 4 as suggested. We have not replicated Table 5, but have instead added the following note that no difference in preferences were observed between participants who had / hadn’t been treated previously for their AS.***

***“No other correlation was found between participant characteristics and MIR/MRB. This includes whether a participant reported having previously undergone treatment for their AS. While this might be expected to influence preferences, the ability of the analysis to identify this influence is limited by the small sample size and the relatively small proportion of participants who reported not having previously received AS treatment (11.8%).”***

***The discussion section has also been updated to include the following statement:***

***“Further research could usefully gather data from a larger sample of AS patients to determine the association of preferences and patient characteristics, such as NYHA classification or whether patients have previously undergone treatment for AS.”***

2. Representativeness of patients involved in member organizations:  The study recruited participants from two member organizations (Heart Valve Voice and Mended Hearts).  While it is understandable why the membership of these organizations facilitated identifying patients with aortic valve disease. However, patients who are motivated enough to join such organizations may have different preferences than the broader population of patients with the disease eligible for treatment of their aortic valve disease.  There is no way to assess this potential difference in this study, but the authors could acknowledge this potential source of bias in the sample population in their discussion of the results and encourage future study in one or more different aortic valve disease populations. 

***Response: We agree that the recruitment strategy may be a source of bias in the preference data. We have acknowledged this in the discussion, which now includes the statement:***

***“***

***Finally, the current study is based on a relatively small sample of patients, and the sample is healthier and younger than the population currently eligible for TAVR and SAVR. AS patients were recruited from the membership of Heart Valve Voice and Mended Hearts, and it is possible that patients who are motivated to join these organizations may have different preferences than the broader population. Further AS patient preference research should replicate this study in a larger sample of patients, including more patients with more severe disease burden”.***

3. Lack of clarity in the attribute of “Time over which the procedure has been proven to work”:  From the definition of this attribute in the paper, it is difficult to know whether patients interpreted this attribute as a measure of how long they could expect benefit, i.e, duration of effect, or whether patients also viewed this as how much clinical experience there was with a treatment, i.e., uncertainty in the knowledge about the effect.  (See section 2 of the MDIC Patient Centered Benefit Risk Report for a nice discussion of uncertainty and how it relates to patient preferences.). This ambiguity raises the question about whether this attribute as described elicited preferences about expected duration, or elicited preferences about patient tolerance of uncertainty about the effect of TAVR, or some combination of the two.  Showing an example of how this “proven to work” attribute was shown to patients akin to Figure 1 might clarify this ambiguity.  Future studies might try to separate these attributes, particularly comparing an established therapy like SAVR with a newer treatment like TAVR. 

***Response: We have updated Figure 1 to use an example of a survey question that includes the ‘time proven to work’  attribute.***

Minor issues and typos:

1. Introduction, last paragraph of left column, p. 3:  the beginning of the second sentence is awkward: “However, little is known about the weights that patients assign to which attributes, . . . . “  I would suggest “However, little is known about the weights that patients assign to each attribute. . . .” ***Response: Updated as suggested***
2. Methods, Attribute selection, last sentence (p.3): note “was were”.  I would remove the were. ***Updated as suggested***
3. Table 1, p. 4: Disabling non-fatal stroke:  in the description, first sentence, note the “one 1” – should be “one month”. ***Response: Updated as suggested***
4. Table 2, p. 4, description of “independence” attribute:  the description of the measure of this attribute does not mention that this is measured at one month, whereas all the other measures note the time frame of the measure. ***Response: The current description includes a timeframe. No update made***
5. Analysis, p. 6, top right column, first paragraph: there looks to be a calculation error in the next to the last sentence.  4.8%/2% = 2.4, not 1.4 as written. ***Response: Tank you for noticing this. Updated. ***
6. Comparisons of TAVR and SAVR, p. 7: right column, top paragraph – “In each case, patents. . . . ” should be “In each case, patients. . . .” ***Response: Updated***
7. Figures 2 and 3, p. 9:  Each x-axis should be labeled with what the unit of measurement is.  This will help the figures be more self-explanatory. ***Response: Labels have been added to the Figures.***
